# Mapping the Functional Assessment of Cancer Therapy - Breast (FACT-B) to the 5-level EuroQoL group’s 5-dimension questionnaire (EQ-5D-5L) utility index in a Multi-ethnic Asian population

**DOI:** 10.1186/s12955-014-0180-6

**Published:** 2014-12-12

**Authors:** Yin Bun Cheung, Nan Luo, Raymond Ng, Chun Fan Lee

**Affiliations:** Center for Quantitative Medicine, Duke-NUS Graduate Medical School, College Road, Singapore, 169857 Singapore; Department of Biostatistics, Singapore Clinical Research Institute, Singapore, Singapore; Department of International Health, University of Tampere, Tampere, Finland; Saw Swee Hock School of Public Health, National University of Singapore, Singapore, Singapore; Department of Medical Oncology, National Cancer Center, Singapore, Singapore

**Keywords:** Breast cancer, EQ-5D-5L, FACT-B, Health utility, Mapping, Quality of life

## Abstract

**Purpose:**

To develop an algorithm for mapping the Functional Assessment of Cancer Therapy – Breast (FACT-B) to the 5-level EuroQoL Group’s 5-dimension questionnaire (EQ-5D-5L) utility index.

**Methods:**

A survey of 238 breast cancer patients in Singapore was conducted. Models using various regression methods with or without recognizing the upper boundary of utility values at 1 were fitted to predict the EQ-5D-5L utility index based on the five subscale scores of the FACT-B. Data from a follow-up survey of these patients were used to validate the results.

**Results:**

A model that maps the physical, emotional, functional well-being and the breast cancer concerns subscales of the FACT-B to the EQ-5D-5L utility index was derived. The social well-being subscale was not associated to the utility index. Although theoretical assumptions may not be valid, ordinary least square outperformed other regression methods. The mean predicted utility index within each performance status level at follow-up deviated from the observed mean less than the minimally important difference of EQ-5D for cancer patients.

**Conclusions:**

The mapping algorithm converts the FACT-B to the EQ-5D utility index. This enables oncologists, clinical researchers and policy makers to obtain a quantitative utility summary of a patient’s health status when only the FACT-B is assessed.

## Introduction

The EuroQoL Group’s 5-dimension questionnaire (EQ-5D) is a generic, preference-based instrument that measures respondents’ health status using a utility index. This interval-type utility index indicates the value of various specific health states using standardized valuation methods such as time trade-off or standard gamble, and is essential in health economic evaluation like cost-utility and quality-adjusted life-year analyses. The EQ-5D is preferred by health technology assessment organisations such as the National Institute for Health and Care Excellence (NICE), United Kingdom, for eliciting health utility values (http://www.nice.org.uk, accessed 6 March 2014). However, preference-based measures are not always used in clinical studies. Most disease-specific instruments are profile-based and only provide ordinal-level measurement scales that cannot be used for health economic analysis. One solution to utilize non-preference-based instruments to perform economic evaluation is to map such measures to the EQ-5D utility index. The NICE has released guidelines and technical support documents to guide the selection and use of mapping algorithms to EQ-5D [1-3]. Since then, there was a substantial increase in the number of studies reporting mapping algorithms to EQ-5D, from one study per year between 2000 and 2003 to 17 studies per year in 2012 and 2013 [4].

The Functional Assessment of Chronic Illness Therapy (FACIT) Measurement System is a collection of health-related quality-of-life (HRQoL) questionnaires for various chronic diseases [5,6]. The core component of the FACIT system is the Functional Assessment of Cancer Therapy – General (FACT-G) for patients of any cancer type. This 27-item instrument can also be extended to a more specific instrument by adding a cancer type specific module. For example, the FACT-G becomes the FACT-Breast (FACT-B) if a 10-item breast cancer-specific module is added. Studies that map the scores of the FACT instruments to the EQ-5D utility index are not rare. Examples include the mapping of FACT-G [7], FACT-Prostate [8] and FACT-Melanoma [9] to the EQ-5D utility index. However, there is no available mapping algorithm in the literature to convert the FACT-B to EQ-5D. Breast cancer has been reported in the Global Cancer Statistics 2011 as the most common type of cancer among women worldwide [10]. In 2008, 1.38 million women were diagnosed of breast cancer accounting for 23% of the total new cancer cases. The purpose of the current study is to formulate a mapping algorithm for generating the EQ-5D health utility index from the FACT-B, so that oncologists and clinical researchers can obtain both a psychometric description and a quantitative utility summary of a patient’s health status from a single assessment using the FACT-B, without imposing additional assessment burden on patients.

## Methods

### Design and recruitment

This is a secondary analysis of a study that aimed to evaluate the measurement properties of the English and Chinese versions of two instruments, namely the FACT-B and the 5-level EQ-5D (EQ-5D-5L), in breast cancer patients in Singapore [11]. The study was approved by the Singapore Health Services Institutional Review Board. Inpatients were recruited from the oncology wards of the Singapore General Hospital while outpatients from the specialist outpatient clinics of the National Cancer Centre, Singapore. Patients who aged 21 years or above, were histologically confirmed breast cancer, able to understand Chinese or English or both, with no evidence of brain metastasis, psychosis or severe depression, and willing to give written informed consent were recruited. They chose to answer either a Chinese or an English questionnaire package according to their preference. Each package included the EQ-5D-5L and FACT-B, together with some questions on demographics and performance status. Approximately one week after the baseline assessment, the patients were sent a similar package with a postage-paid return envelope enclosed to evaluate their HRQoL, health utility and performance status for assessment of test-retest reliability of the instruments. Up to two reminders with the questionnaire package were sent at two-weekly intervals if the package was not returned. Other clinical information was provided by the patients’ treating oncologists. At baseline, the questionnaire package was self-administered by the patients, or by a research assistant upon patient’s request. At follow-up, it was self-administered by the patients. Questionnaires not self-administered were excluded from this analysis.

### Instruments and variables

The FACT-B is a breast cancer-specific HRQoL instrument of the FACIT system. The 37-item English and (simplified) Chinese FACT-B version 4 are divided into five subscales, namely physical (PWB), social/family (SWB), emotional (EWB), functional well-beings (FWB), and the additional concerns for breast cancer (BCS) [12,13]. We have reported the validity and reliability of, and the comparability between the two language versions in an earlier study [14]. Each item is rated on a 5-point Likert scale. Negatively worded items were recoded such that a higher score indicates a better HRQoL. The FACT-B total score is the sum of scores of all five subscales, the FACT-G score is the sum of PWB, SWB, EWB and FWB, while the Trial Outcome Index (TOI) is the sum of scores of the PWB, FWB and BCS. Missing values were imputed as the mean of observed items provided more than half of the items comprising a subscale were answered, i.e. the “half-rule” [5].

The EQ-5D-5L contains five questions (mobility, self-care, usual activities, pain/discomfort, and anxiety/depression), plus a vertical, 0-100-point visual analogue scale for rating the overall health status. Respondents could choose one of the five levels to describe their health state on the day of survey. These five levels include “no problem”, “slight problems”, “moderate problems” and “severe problems” in all five dimensions, and “unable” in mobility, self-care and usual activities or “extreme problems” in pain/discomfort and anxiety/depression [15]. In this study, experimental English and Chinese versions of the EQ-5D-5L were used because the official version was not available at the time. The differences between the official and experimental versions are minor [16]. In the official English version the responses start with “I have” or “I am,” which are omitted in the experimental English version. For the self-care dimension, the official Chinese version asks the degree of problems in “washing and dressing” while the experimental Chinese version asks about “combing, washing and dressing.” Other differences in Chinese version only involve the use of some words which are actually synonyms and commonly used in daily life, e.g., the English word “usual” is translated to “*ri chang*” (in Chinese phonetic transcription here) in the official Chinese version and “*pin chang*” in the experimental version. Recently, we reported the validity and reliability of, and the comparability between the English and Chinese versions of the EQ-5D-5L [16].

The answers to the same five questions in an older 3-level version, EQ-5D-3 L, can be converted to a utility index through some country-specific value set. For the conversion of the EQ-5D-5L to a utility index, however, an official value set has not been released by the EuroQoL Group at the time of writing this report. Instead, a crosswalk project converting the EQ-5D-3 L value set to the EQ-5D-5L was conducted by the EuroQoL Group which resulted in the interim value sets [17]. We obtained and used the Japanese value set (Dr. Rosalind Rabin, the EuroQoL Group), the only Asian value set available in the crosswalk project. Using this value set, the EQ-5D-5L utility index has a possible range of −0.111 to 1 [18]. As a sensitivity analysis, the UK value with possible range of −0.594 to 1 was also employed. A higher index means a better health state, with 1, 0, positive values between 1 and 0, and negative values corresponding to full health, death, health states better than death but worse than full health, and health states worse than death, respectively.

The performance status is known to be strongly associated with patients’ quality of life [19], and can be assessed by both the oncologists and the patients themselves [20]. Respondents choose the most appropriate one that describes the cancer patient from five options ranging from 0 (without symptoms) to 4 (bedridden) [21]. The score of 5 (death) was not applicable in this study.

### Statistical analysis

The baseline survey was used for developing the mapping functions. The EQ-5D-5L utility index was regressed on a combination of the five subscales of the FACT-B. Five different models were examined. Model 1 included all five subscales of the FACT-B, Model 2 the four subscales of the FACT-G, and Model 3 the three subscales of the TOI. Based on some preliminary analysis, the coefficients of the SWB subscale in Models 1 and 2 were found to be insignificant and small in magnitude, and in some cases negative. Therefore, two more models (Models 4 and 5) were conducted by dropping the SWB subscale. Since equivalence between English and Chinese versions of the two instruments could not be surely confirmed in the previous studies [14,16], we also conducted some sensitivity analyses by fitting models that included language as well as interactions between language and the subscales as predictors to investigate whether it should be included in the mapping algorithm.

The EQ-5D-5L utility index is bounded from above by 1 which indicates full health. Previous studies pointed out that this upper bound may invalidate the normality assumption of ordinary least square (OLS) method. Tobit model, an alternative to OLS to deal with censored data, has been suggested for use in mapping. However, it is inconsistent to the presence of heteroscedasticity [22,23]. Censored least absolute deviations (CLAD) is a solution to the heteroscedasticity problem in the Tobit model [24]. This has also been used in the current context. However, an upper bound is not the same as a censoring threshold. Thus, apart from OLS, Tobit and CLAD models, we also considered two regression methods, namely quantile regression and logistic quantile regression [25,26]. Quantile regression is also called median regression if the second quantile, i.e. median, is fitted (as in this study) [25]. The difference between CLAD and median regression is that the former assumes the dependent variable is a censored value of some unobserved latent variable, while the latter does not. In other words, CLAD is a censored version of median regression. Logistic quantile regression is an approach for handling bounded outcomes [26], which is theoretically correct for mapping of utility values. Suppose the outcome *y* is bounded from below and from above by two known constants, *y*_*min*_ and *y*_*max*_, respectively. A logistic transform is applied to the outcome to obtain

$$ h(y)= log\left(\frac{y-{y}_{min}}{y_{max}-y}\right), $$

and a quantile regression is then fitted by regressing the transformed outcome *h*(*y*) on the independent variables. In practice, to ensure the logistic transform is defined for all observed values, *y*_*min*_ is set to be slightly smaller than the smallest observed outcome, and *y*_*max*_ slightly larger than the largest observed outcome. In this study, we set *y*_*min*_ = 0.17885 for Japanese value set and −0.28265 for UK value set (i.e. half of the observed smallest increment less than the smallest observed utility value from the respective value set [27]), and *y*_*max*_ = 1.001 for both value sets. The visual analogue scale of the EQ-5D was not used in the analysis.

Model performance was examined by several goodness-of-fit measures. The coefficient of determination, *R*^2^, in OLS may not be well-defined in other regression methods. The pseudo-*R*^2^, an alternative to *R*^2^ defined by the likelihood ratio between the intercept-only model and the full model, is not comparable across different regression methods [28]. Instead, we computed the square of the correlation coefficient (*r*) between the observed and predicted values from each model. Note that *R*^2^ is equivalent to *r*^2^ in OLS. Parallel to OLS, to penalize for the complexity of the model, we considered an adjusted *r*^2^ defined as

$$ \mathrm{adjusted}\ {r}^2 = 1\ \hbox{--} \frac{\left(n\ \hbox{--}\ 1\right)\ }{\left(n\ \hbox{--}\ p\ \hbox{--}\ 1\right)}\left(1\ \hbox{--}\ {r}^2\right), $$

where *n* is the sample size and *p* is the number of parameters in the model. Goodness-of-fit was also examined by mean square error (MSE) and mean absolute deviation (MAD). Because OLS minimizes the sum of squared deviations whereas CLAD and quantile regression minimize the sum of absolute deviations, one would expect that the MSE tends to favor the OLS while MAD tends to favor the CLAD and quantile regression. Therefore, the interpretation should not focus exclusively on one index. The distributions of the observed and mapped utility values were also compared. The follow-up survey was used for validating the resulted algorithms. The differences between the observed and predicted utility values at follow-up were tested by signed-rank tests within each performance status level. All statistical analyses were performed in SAS system version 9.3 (SAS Institute, Cary, NC, USA) and Stata version 10 (StatCrop, College Station, TX).

## Results

Two hundred and eighty female breast cancer patients consented to participate and answered the questionnaire package. At baseline, 39 patients did not self-administer the questionnaire package and were thus removed from the analysis. Three patients were further excluded due to missing values in the EQ-5D-5L or FACT-B beyond imputation by the half-rule. As a result, 238 patients at baseline were used in the development sample. Their demographic and clinical information at baseline is summarized in Table 1. Most of the patients answered an English package (67.2%), were ethnic Chinese (81.1%), married (70.9%) and outpatients (70.6%). Among them, 221 returned the questionnaire package with no missing values, and were used for validation.

**Table 1:** **Demographic and baseline information of 238 eligible subjects**

**Characteristics**	**N**	**(%)**
Age (year), Mean (standard deviation)	51.3	(9.7)
Language of questionnaire		
English	160	(67.2)
Chinese	78	(32.8)
Race		
Chinese	193	(81.1)
Malay	23	(9.7)
Indian	18	(7.6)
Others	4	(1.7)
Marital status		
Married	168	(70.9)
Single	47	(19.8)
Divorced/separated	12	(5.1)
Widowed	10	(4.2)
Education level		
Primary or below	48	(20.3)
Secondary	112	(47.3)
Postsecondary	77	(32.5)
Inpatient/Outpatient		
Inpatient	70	(29.4)
Outpatient	168	(70.6)
Patient-assessed performance status		
0	97	(40.8)
1	115	(48.3)
2	17	(7.1)
3 or 4	9	(3.8)
Evidence of disease		
Present	118	(50.0)
Absent	118	(50.0)
Purpose of visit		
Treatment – Adjuvant/curative/hormone therapy	112	(47.7)
Treatment – Palliative	78	(33.2)
No treatment – Follow-up	45	(19.1)
On chemotherapy/radiotherapy		
Yes	100	(42.0)
No	138	(58.0)

Table 2 describes the distributions of the EQ-5D-5L utility index and the FACT-B total and subscale scores of the sample at baseline and follow-up. There were approximately a quarter of patients reporting full EQ-5D-5L utility value at baseline and one fifth at follow-up. No patient reported a maximum FACT-B total score at both time points, but the FACT-G score and four subscales (PWB, SWB, EWB and FWB) reached the upper bound in some patients. For the BCS subscale, one patient had maximum score at baseline, but none at follow-up.

**Table 2:** **Distribution of EQ-5D-5L and FACT-B scores and subscales at baseline and follow-up**

**Observed Scores**	**Mean**	**SD**	**Min**	**Q1**	**Median**	**Q3**	**Max**	**Lower bound**	**Upper bound**
*Baseline (N = 238)*									
EQ-5D-5L utility index									
Japanese value set	0.777	0.163	0.179	0.686	0.740	0.843	1.000	0 (0.0%)	59 (24.8%)
UK value set	0.785	0.200	−0.283	0.721	0.777	0.906	1.000	0 (0.0%)	59 (24.8%)
FACT-B									
Total score	103.0	20.8	45	90	104	119	140	0 (0.0%)	0 (0.0%)
FACT-G score	81.3	16.9	35	71	83	95	108	0 (0.0%)	4 (1.7%)
Trial outcome index	62.8	14.9	16	53	64	74	89	0 (0.0%)	0 (0.0%)
Physical well-being	21.1	6.0	1	18	22	26	28	0 (0.0%)	29 (12.2%)
Social well-being	22.2	6.1	0	20	24	27	28	3 (1.3%)	50 (21.0%)
Emotional well-being	18.0	4.7	4	15	19	22	24	0 (0.0%)	28 (11.8%)
Functional well-being	20.0	6.7	0	16	21	26	28	3 (1.3%)	31 (13.0%)
Breast cancer subscale	21.7	6.1	5	17	22	27	36	0 (0.0%)	1 (0.4%)
*Follow-up (N = 221)*									
EQ-5D-5L utility index									
Japanese value set	0.758	0.160	0.052	0.649	0.740	0.829	1.000	0 (0.0%)	46 (20.8%)
UK value set	0.770	0.193	−0.283	0.679	0.768	0.879	1.000	0 (0.0%)	46 (20.8%)
FACT-B									
Total score	101.2	22.2	27	85	103	118	140	0 (0.0%)	0 (0.0%)
FACT-G score	79.6	18.0	20	67	82	94	108	0 (0.0%)	5 (2.3%)
Trial outcome index	61.6	15.8	9	51	63	74	89	0 (0.0%)	0 (0.0%)
Physical well-being	21.0	6.1	0	17	22	26	28	2 (0.9%)	26 (11.8%)
Social well-being	21.7	5.5	4	18	22	27	28	0 (0.0%)	40 (18.1%)
Emotional well-being	17.9	4.7	3	15	19	21	24	0 (0.0%)	25 (11.3%)
Functional well-being	19.0	6.9	2	14	21	25	28	0 (0.0%)	20 (9.0%)
Breast cancer subscale	21.6	6.2	7	17	22	26	35	0 (0.0%)	0 (0.0%)

*Abbreviations:* *SD* standard deviation, *Min* minimum, *Q1* 1^st^ quartile, *Q3* third quartile, *Max* maximum.

The results of the regression analyses using the Japanese value set are displayed in Table 3. For each regression method, five models were fitted. Among the five models, Model 4 consisting of the PWB, EWB, FWB and BCS had the largest adjusted *r*^2^, regardless of the regression method used. The MSE and MAD of the five models were similar, but mostly Model 4 had the smallest, with the only exception in the MAD (0.0913) which was marginally larger than that of Model 1 (0.0912) for CLAD and quantile regression. Among the five regression methods, OLS generally had the largest adjusted *r*^2^ (0.4782 to 0.4887) and smallest MSE (0.0132 to 0.0135) and MAD (0.0913 to 0.0925), followed by CLAD and quantile regression (adjusted *r*^2^ ranged from 0.4775 to 0.4882; MSE from 0.0133 to 0.0135; MAD from 0.0912 to 0.0923). The latter two had the same point estimates for the regression coefficients in all models. However, due to different model assumption and estimation process, their standard errors were not the same (details not shown), and hence the p-values were different.

**Table 3:** **Coefficient estimates and goodness-of-fit measures of various regression models mapping the FACT-B subscales to EQ-5D-5L utility index based on baseline survey (N = 238)**

	**Model 1**	**Model 2**	**Model 3**	**Model 4**	**Model 5**
*Ordinary least square*					
*Regression coefficients*					
Constant	0.2901^**^	0.3071^**^	0.3064^**^	0.2846^**^	0.3031^**^
Physical well-being	0.0120^**^	0.0127^**^	0.0125^**^	0.0121^**^	0.0128^**^
Social well-being	−0.0003	−0.0003			
Emotional well-being	0.0042^*^	0.0062^**^		0.0041	0.0061^**^
Functional well-being	0.0046^**^	0.0048^**^	0.0052^**^	0.0044^**^	0.0046^**^
Breast cancer subscale	0.0035^*^		0.0048^**^	0.0034^*^	
*Goodness-of-fit measures*					
Square of correlation coefficient, *r* ^2^	0.4975	0.4870	0.4890	0.4973	0.4870
Adjusted *r* ^2^	0.4867	0.4782	0.4824	0.4887	0.4804
Mean square error	0.0132	0.0135	0.0135	0.0132	0.0135
Mean absolute deviation	0.0913	0.0918	0.0925	0.0913	0.0919
*Tobit model*					
*Regression coefficients*					
Constant	0.1983^**^	0.2200^**^	0.2342^**^	0.2077^**^	0.2318^**^
Physical well-being	0.0144^**^	0.0153^**^	0.0147^**^	0.0142^**^	0.0152^**^
Social well-being	0.0006	0.0007			
Emotional well-being	0.0050	0.0077^**^		0.0051	0.0078^**^
Functional well-being	0.0048^*^	0.0051^**^	0.0060^**^	0.0051^**^	0.0054^**^
Breast cancer subscale	0.0045^*^		0.0063^**^	0.0046^*^	
*Goodness-of-fit measures*					
Square of correlation coefficient, *r* ^2^	0.4965	0.4861	0.4887	0.4971	0.4869
Adjusted *r* ^2^	0.4856	0.4773	0.4821	0.4885	0.4803
Mean square error	0.0143	0.0146	0.0145	0.0143	0.0145
Mean absolute deviation	0.0934	0.0937	0.0945	0.0934	0.0936
*Censored least absolute deviation*					
*Regression coefficients*					
Constant	0.3062^**^	0.3208^**^	0.3200^**^	0.2948^**^	0.3149^**^
Physical well-being	0.0119^**^	0.0121^**^	0.0123^**^	0.0122^**^	0.0121^**^
Social well-being	−0.0004	−0.0003			
Emotional well-being	0.0041	0.0054^**^		0.0042	0.0054^**^
Functional well-being	0.0037^*^	0.0054^**^	0.0039^*^	0.0037^*^	0.0053^**^
Breast cancer subscale	0.0036		0.0054^**^	0.0035	
*Goodness-of-fit measures*					
Square of correlation coefficient, *r* ^2^	0.4968	0.4863	0.4873	0.4968	0.4862
Adjusted *r* ^2^	0.4860	0.4775	0.4807	0.4882	0.4796
Mean square error	0.0133	0.0135	0.0135	0.0133	0.0135
Mean absolute deviation	0.0912	0.0917	0.0923	0.0913	0.0917
	**Model 1**	**Model 2**	**Model 3**	**Model 4**	**Model 5**
*Quantile regression*					
*Regression coefficients*					
Constant	0.3062^**^	0.3208^**^	0.3200^**^	0.2948^**^	0.3149^**^
Physical well-being	0.0119^**^	0.0121^**^	0.0123^**^	0.0122^**^	0.0121^**^
Social well-being	−0.0004	−0.0003			
Emotional well-being	0.0041	0.0054^**^		0.0042^*^	0.0054^**^
Functional well-being	0.0037^**^	0.0054^**^	0.0039^**^	0.0037^**^	0.0053^**^
Breast cancer subscale	0.0036		0.0054^**^	0.0035	
*Goodness-of-fit measures*					
Square of correlation coefficient, *r* ^2^	0.4968	0.4863	0.4873	0.4968	0.4862
Adjusted *r* ^2^	0.4860	0.4775	0.4807	0.4882	0.4796
Mean square error	0.0133	0.0135	0.0135	0.0133	0.0135
Mean absolute deviation	0.0912	0.0917	0.0923	0.0913	0.0917
*Logistic quantile regression*					
*Regression coefficients*					
Constant	−1.6525	−1.6097	−1.5750^*^	−1.6489	−1.6678
Physical well-being	0.0684	0.0710	0.0678^*^	0.0683^**^	0.0722^**^
Social well-being	0.0001	−0.0035			
Emotional well-being	0.0155	0.0320		0.0157	0.0308
Functional well-being	0.0230	0.0312	0.0247	0.0230	0.0295
Breast cancer subscale	0.0216		0.0301	0.0214	
*Goodness-of-fit measures*					
Square of correlation coefficient, *r* ^2^	0.4686	0.4596	0.4620	0.4687	0.4597
Adjusted *r* ^2^	0.4571	0.4503	0.4551	0.4596	0.4528
Mean square error	0.0141	0.0143	0.0143	0.0141	0.0143
Mean absolute deviation	0.0946	0.0949	0.0953	0.0946	0.0950

^**^P-value < 0.01; ^*^P-value < 0.05.

In each of the models that include language and/or interactions between language and the subscales as predictors, the coefficient estimates were small in magnitude and insignificant. Moreover, adding language into the model did not improve the goodness-of-fit nor alter qualitatively the parameter estimates of other variables. Models with interaction and quadratic terms of the subscales were also fitted. However, these terms were insignificant and did not improve the model in terms of goodness-of-fit (details not shown). Moreover, the results using the UK value set were similar to that of the Japanese value set, hence were not shown here either. One point worth noting is that CLAD and quantile regression had different point estimates when the UK value set was used.

The observed and predicted EQ-5D-5L utility index values by Models 3 through 5 at baseline survey were compared (Table 4). For central tendency, the five methods performed differently. As restricted by the estimation process, the means of the predicted values based on OLS were always the same as that of the observed values; but the median of the predicted values were larger than that of the observed ones. The Tobit models tended to produce larger predicted values than the observed ones, hence resulting in larger means and medians. CLAD, quantile regression and logistic quantile regression had the mean predicted values slightly smaller than the observed mean, but the medians were also larger than the observed median. For dispersion, however, predicted values by all methods had a smaller spread than the observed values. Compared with the observed values, the predicted values by each model had a smaller standard deviation, larger minimum and 10^th^ percentile but smaller 90^th^ percentile and maximum. It was possible that the predicted EQ-5D-5L utility index fell outside the defined range of −0.111 to 1 (for Japanese value set). However, this only happened in less than 3% of the sample in Models 3 and 4 when the Tobit method was used.

**Table 4:** **Descriptive summary of the baseline EQ-5D-5L utility index derived from observed data and regression models**

**Model**	**Mean**	**SD**	**Min**	**P10**	**Q1**	**Median**	**Q3**	**P90**	**Max**	**Upper bound**
Observed data	0.7772	0.1626	0.1789	0.6079	0.6856	0.7402	0.8428	1.0000	1.0000	24.8%
*Ordinary least square*										
Model 3	0.7772	0.1137	0.3930	0.6162	0.7059	0.7902	0.8643	0.9150	0.9583	0%
Model 4	0.7772	0.1147	0.3830	0.6149	0.7082	0.7898	0.8648	0.9146	0.9498	0%
Model 5	0.7772	0.1135	0.3868	0.6211	0.7100	0.7891	0.8660	0.9138	0.9394	0%
*Tobit model*										
Model 3	0.8005	0.1361	0.3414	0.6101	0.7127	0.8185	0.9032	0.9659	1.0203	2.5%
Model 4	0.8004	0.1372	0.3292	0.6057	0.7165	0.8153	0.9059	0.9653	1.0092	2.9%
Model 5	0.8004	0.1355	0.3341	0.6126	0.7224	0.8158	0.9059	0.9635	0.9949	0%
*Censored least absolute deviation*										
Model 3	0.7761	0.1095	0.4044	0.6274	0.7102	0.7887	0.8573	0.9094	0.9531	0%
Model 4	0.7760	0.1119	0.3896	0.6188	0.7119	0.7879	0.8609	0.9099	0.9448	0%
Model 5	0.7743	0.1107	0.3965	0.6183	0.7070	0.7870	0.8602	0.9083	0.9326	0%
*Quantile regression*										
Model 3	0.7761	0.1095	0.4044	0.6274	0.7102	0.7887	0.8573	0.9094	0.9531	0%
Model 4	0.7760	0.1119	0.3896	0.6188	0.7119	0.7879	0.8609	0.9099	0.9448	0%
Model 5	0.7743	0.1107	0.3965	0.6183	0.7070	0.7870	0.8602	0.9083	0.9326	0%
*Logistic quantile regression*										
Model 3	0.7691	0.1042	0.3861	0.6221	0.7141	0.7936	0.8469	0.8804	0.9038	0%
Model 4	0.7680	0.1051	0.3780	0.6153	0.7146	0.7896	0.8463	0.8799	0.8971	0%
Model 5	0.7675	0.1085	0.3687	0.6093	0.7104	0.7914	0.8506	0.8817	0.8957	0%

*Abbreviations:* *SD* standard deviation, *Min* minimum, *P10* 10^th^ percentile, *Q1* 1^st^ quartile, *Q3* third quartile, *P90* 90^th^ percentile, *Max* maximum.

Table 5 presents the mean observed and predicted EQ-5D-5L utility index by Models 3 through 5 classified by performance status at follow-up. The utility index was significantly overestimated in the group with performance status of 1, but underestimated in the group with performance status of 0 only when the logistic quantile regression was used. The largest differences between observed and predicted values were approximately 0.06. The predicted utility index showed significantly decreasing trend with performance status (all p-values for trend < 0.001).

**Table 5:** **Mean EQ-5D-5L utility index by patients’ self-assessed performance status at follow-up**

	**Performance status**				**P-value for trend** ^**a**^
**Model**	**0**	**1**	**2**	**3 or 4**	
Observed data	0.8719	0.7273	0.5941	0.5941	
*Ordinary least square*					
Model 3	0.8518	0.7584^**^	0.6499	0.5886	< 0.001
Model 4	0.8537	0.7581^**^	0.6518	0.5883	< 0.001
Model 5	0.8560	0.7571^**^	0.6495	0.5842	< 0.001
*Tobit model*					
Model 3	0.8890	0.7784^**^	0.6498	0.5772	< 0.001
Model 4	0.8913	0.7780^**^	0.6520	0.5768	< 0.001
Model 5	0.8944	0.7766^**^	0.6490	0.5710	< 0.001
*Censored least absolute deviation*					
Model 3	0.8478^*^	0.7590^**^	0.6558	0.6004	< 0.001
Model 4	0.8512	0.7580^**^	0.6545	0.5948	< 0.001
Model 5	0.8505	0.7541^**^	0.6487	0.5829	< 0.001
*Quantile regression*					
Model 3	0.8478^*^	0.7590^**^	0.6558	0.6004	< 0.001
Model 4	0.8512	0.7580^**^	0.6545	0.5948	< 0.001
Model 5	0.8505	0.7541^**^	0.6487	0.5829	< 0.001
*Logistic quantile regression*					
Model 3	0.8341^**^	0.7548^**^	0.6526	0.5920	< 0.001
Model 4	0.8349^**^	0.7532^**^	0.6507	0.5886	< 0.001
Model 5	0.8387^**^	0.7508^**^	0.6422	0.5733	< 0.001

^a^Cuzick’s nonparametric test for trend [29].

^**^P-value < 0.01; ^*^P-value < 0.05 for signed-rank test between observed and predicted utility index within the same performance status level.

## Discussion

The regression analyses showed that the EQ-5D-5L index of the breast cancer patients in our sample was best predicted by the model consisting of four FACT-B subscales, i.e., PWB, EWB, FWB and BCS (Model 4). Although the coefficients of BCS and EWB were respectively significant in Model 3 and Model 5 for most regression methods, they were not both significant in Model 4. However, this study aimed to construct a model that best predicts the utility index, so whether the regression coefficients are statistically significant is of secondary consideration. The most important criterion is the accuracy of the prediction. The observed and predicted values correlated more closely in Model 4 than in Models 3 and 5 even after penalizing for model complexity, and the MSE and MAD were the smallest in Model 4. Although BCS contains several emotion-related items, those items are more specific to breast cancer patients than those in the EWB subscales, e.g., “*I feel sexually attractive,*” “*I am able to feel like a woman*”, etc. Therefore, it is appropriate to include both BCS and EWB in the model. On the other hand, due to a lack of social-related item in the EQ-5D-5L, SWB was not associated with the utility index. In the current study and two previous mapping studies of FACT-G and FACT-Prostate, the coefficient for SWB was negative despite being insignificant [7,8]. Although this is counter-intuitive as one would not expect that better social well-being leads to lower health utility, it is possible that patients in declining health perceive social-related quality-of-life more positively than those in better health.

In this study, OLS had the best goodness-of-fit while the performance of CLAD and quantile regression were close to OLS, but Tobit model and logistic quantile regression did not perform well. Previous studies showed that CLAD and OLS were superior to Tobit model in developing a mapping algorithm to the EQ-5D utility index, but whether CLAD or OLS performed better, or in what situation (e.g. proportion of patients attaining full health) one method outperformed the other was inconclusive [28,30,31]. The OLS approach has some merits that are not shared by other methods, such as well-developed diagnostic checking techniques and its availability in common statistical packages. In fact, as reported in two recent reviews, OLS was the most commonly used method in mapping studies [4,32]. Both reviews, however, were concerned by the quality of these mapping algorithms using OLS. Hence, further research on the usability of OLS in mapping studies is warranted.

Similar to our findings from the Japanese value set, Austin et al. also obtained identical point estimates in CLAD and quantile regression [22]. It has been pointed out that Tobit and CLAD models should not be used for analyzing health utility index because it is conceptually bounded from above by 1, rather than censored at 1 [24]. In contrast, quantile regression recognizes the data truly cannot exceed the upper boundary, as opposed to assuming censoring at the boundary. Therefore, quantile regression is conceptually more appropriate than Tobit or CLAD. Logistic quantile regression, although not performing well in the current study, has a theoretical advantage that the logistic transform can deal with the boundary problem [26]. This not only suits the distributional property of health utility index, but also guarantees the predicted values would not exceed the boundary of 1.

The *r*^2^ values were no larger than 0.5 for all models (Table 3), implying that at least half of the variance in the data has not been accounted for. This moderate level of accuracy is not uncommon in studies mapping FACT instruments to EQ-5D utility index, e.g., *R*^2^ ranged from 0.317 to 0.451 for FACT-G [7], from 0.535 to 0.582 for FACT-Prostate [8], and from 0.328 to 0.499 for FACT-Melanoma [9]. On the other hand, the models were also estimated in terms of the mean utility index in patients with different level of performance status at follow-up. Some of the differences between the mean observed and predicted values within the same level were found statistically significant. However, the differences were small and less than the minimally important differences of EQ-5D for cancer patients (0.08 for UK value set and 0.06 US value set) [33]. Thus we believed that the differences were clinically unimportant. Furthermore, the mean predicted values showed a clear trend in relation to the performance status, suggesting their good discriminating ability. Hence, the mapping algorithm appears to be appropriate for use at group level and may not be so at individual level.

We also tried mapping the individual items, instead of subscales, of the FACT-B to EQ-5D-5L utility index using OLS with stepwise selection. Although the model using items as predictors obtained comparable *r*^2^, MSE and MAD to that of Model 4 using OLS in Table 3, this model resulted in a smaller adjusted *r*^2^ due to a larger number of predictors, which implies a more complex model. More importantly, in practice, missing values can be imputed by the “half-rule” if subscales are being mapped to utility index, but may not be properly handled if individual items are used. Another possibility to derive a FACT-B preference-based score is direct valuation. However, unlike the EQ-5D that has only 5 items, FACT-B has 37 items that makes direct valuation difficult due to the large number of possible health states. Therefore, it is practically not feasible.

A limitation of this study is that the value set for the 5-level EQ-5D instrument was based on a crosswalk project that linked the EQ-5D-3 L to the EQ-5D-5L [17]. Some recently published studies have reported the value set of several Asian countries for EQ-5D-3 L but not for EQ-5D-5L. The EQ-5D-5L value sets are best to be estimated through direct valuation of the health states using methods such as time trade-off. Nevertheless, this crosswalk algorithm is currently the only available one for converting the EQ-5D-5L responses to a utility index, and the results are robust as indicated by a sensitivity analysis using the UK value set. In fact, both Japan and Singapore are highly modernized and their well-educated populations are exposed to international influence. In a review study of the development of HRQoL instruments in Asian languages, Cheung and Thumboo found that QoL concepts do not vary a lot with cultures, and items that are highly specific to a society are uncommon [34]. Sakthong et al. compared the psychometric properties among 3 value sets of the EQ-5D-3 L using a sample of individuals from Thailand, another south-east Asian country, and revealed that the Japanese value set obtained better test-retest reliability, convergent validity and known-group validity than the UK and US value sets [35]. Therefore, the use of the Japanese value set, though not ideal, is expected to be appropriate for Singapore. That said, our findings are best verified when there is a local value set available in the future. Moreover, nearly 90% of the patients in this study had a performance status of 0 to 1. This may limit the generalizability of the results to more severe patients. Furthermore, instead of answering both English and Chinese versions of the instruments, bilingual subjects only selected the language they preferred to use. This limited head-to-head comparison between the language versions and assessment of equivalence. That said, survey language was not significantly associated with the EQ-5D-5L utility index and did not improve the goodness-of-fit nor qualitatively influence the coefficient estimates of other independent variables, suggesting that pooling the data from English and Chinese versions for analysis is reasonable.

In conclusion, this study confirmed the feasibility of mapping the FACT-B subscale scores to the EQ-5D-5L utility index. Among the various regression methods tested, OLS not only provided the best goodness-of-fit measures, but also has the widest availability of statistical packages for analysis and diagnostic checking. Hence, we recommend OLS for mapping the FACT-B to the EQ-5D-5L utility index. The findings are useful in cost-utility and quality-adjusted life-year analyses for breast cancer patients when only the FACT-B data are available.

## References

[1] National Institute for Health and Clinical Excellence: **Guide to the methods of technology appraisal 2013.** Available at https://www.nice.org.uk/article/pmg9/resources/non-guidance-guide-to-the-methods-of-technology-appraisal-2013-pdf. accessed 13 December 2014. 27905712

[2] Longworth L, Rowen D (2011). NICE DSU Technical Support Document 10: the Use of Mapping Methods to Estimate Health State Utility Values.

[3] Longworth L, Rowen D (2013). Mapping to obtain EQ-5D utility values for use in NICE health technology assessments. Value Health.

[4] Dakin H (2013). Review of studies mapping from quality of life or clinical measures to EQ-5D: an online database. Health Qual Life Outcomes.

[5] Cella D (1997). FACIT Manual: Manual of the Functional Assessment of Chronic Illness Therapy (FACIT) Measurement System.

[6] Webster K, Cella D, Yost K (2003). The Functional Assessment of Chronic Illness Therapy (FACIT) measurement system: properties, applications, and interpretation. Health Qual Life Outcomes.

[7] Cheung YB, Thumboo J, Gao F, Ng GY, Pang G, Koo WH, Sethi VK, Wee J, Goh C (2009). Mapping the English and Chinese versions of the functional assessment of cancer therapy-general to the EQ-5D utility index. Value Health.

[8] Wu EQ, Mulani P, Farrell MH, Sleep D (2007). Mapping FACT-P and EORTC QLQ-C30 to patient health status measured by EQ-5D in metastatic hormone-refractory prostate cancer patients. Value Health.

[9] Askew RL, Swartz RJ, Xing Y, Cantor SB, Ross MI, Gershenwald JE, Palmer JL, Lee JE, Cormier JN (2011). Mapping FACT-melanoma quality-of-life scores to EQ-5D health utility weights. Value Health.

[10] Jemal A, Bray F, Center MM, Ferlay J, Ward E, Forman D (2011). Global cancer statistics. CA Cancer J Clin.

[11] Lee CF, Luo N, Ng R, Wong NS, Yap YS, Lo SK, Chia WK, Yee A, Krishna L, Wong C, Goh C, Cheung YB (2013). Comparison of the Measurement properties between a short and generic instrument, the 5-level EuroQoL Group’s 5-Dimension (EQ-5D-5L) questionnaire, and a longer and disease-specific instrument, the Functional Assessment of Cancer Therapy – Breast (FACT-B), in Asian breast cancer patients. Qual Life Res.

[12] Wan C, Zhang D, Yang Z, Tu X, Tang W, Feng C, Wang H, Tang X (2007). Validation of the simplified Chinese version of the FACT-B for measuring quality of life for patients with breast cancer. Breast Cancer Res Treat.

[13] Brady M, Cella DF, Mo F, Bonomi AE, Tulsky DS, Lloyd SR, Deasy S, Cobleigh M, Shiomoto G (1997). Reliability and validity of the functional assessment of cancer therapy – breast quality-of-life instrument. J Clin Oncol.

[14] Ng R, Lee CF, Wong NS, Luo N, Yap YS, Lo SK, Chia WK, Yee A, Krishna L, Goh C, Cheung YB (2012). Measurement properties of the English and Chinese versions of the Functional Assessment of Cancer Therapy – Breast (FACT-B) in Asian breast cancer patients. Breast Cancer Res Treat.

[15] Pickard AS, Kohlmann T, Janssen MF, Bonsel G, Rosenbloom S, Cella D (2007). Evaluating equivalency between response systems application of the Rasch model to a 3-level and 5-level EQ-5D. Med Care.

[16] Lee CF, Ng R, Luo N, Wong NS, Yap YS, Lo SK, Chia WK, Yee A, Krishna L, Wong C, Goh C, Cheung YB (2013). The English and Chinese versions of the 5-level EuroQoL Group’s 5-dimension questionnaire (EQ-5D) were valid and reliable and provided comparable scores in Asian breast cancer patients. Support Care Cancer.

[17] van Hout B, Janssen MF, Feng YS, Kohlmann T, Busschbach J, Golicki D, Lloyd A, Scalone L, Kind P, Pickard AS (2012). Interim scoring for the EQ-5D-5L: mapping the EQ-5D-5L to EQ-5D-3 L value sets. Value Health.

[18] Tsuchiya A, Ikeda S, Ikegami N, Nishimura S, Sakai I, Fukuda T, Hamashima C, Hisashige A, Tamura M (2002). Estimating an EQ-5D population value set: the case of Japan. Health Econ.

[19] Cheung YB, Goh C, Thumboo J, Khoo KS, Wee J (2005). Variability and sample size requirements of quality-of-life measures: a randomized study of three major questionnaires. J Clin Oncol.

[20] Blagden SP, Charman SC, Sharples LD, Magee LR, Gilligan D (2003). Performance status score: do patients and their oncologists agree?. Br J Cancer.

[21] Taylor AE, Olver IN, Sivanthan T, Chi M, Purnell C (1999). Observer error in grading performance status in cancer patients. Support Care Cancer.

[22] Austin PC (2002). A comparison of methods for analyzing health-related quality-of-life measures. Value Health.

[23] Powell JL (1984). Least absolute deviations estimation for the censored regression model. J Econometrics.

[24] Pullenayegum EM, Tarride JE, Xie F, Goeree R, Gerstein HC, O’Reilly D (2010). Analysis of health utility data when some subjects attain the upper bound of 1: Are Tobit and CLAD models appropriate?. Value Health.

[25] Austin PC, Tu JV, Daly PA, Alter DA (2005). The use of quantile regression in health care research: a case study examining gender differences in the timeliness of thrombolytic therapy. Stat Med.

[26] Bottai M, Cai B, McKeown RE (2010). Logistic quantile regression for bounded outcomes. Stat Med.

[27] Orsini N, Bottai M (2011). Logistic quantile regression in Stata. Stata J.

[28] Sullivan PW, Ghushchyan V (2006). Mapping the EQ-5D index from the SF-12: US geneneral population preferences in a nationally representative sample. Med Decis Making.

[29] Cuzick J (1985). A Wilcoxon-type test for trend. Stat Med.

[30] Chuang LH, Kind P (2009). Converting the SF-12 into the EQ-5D: an empirical comparison of methodologies. Pharmacoeconomics.

[31] Cheung YB, Tan LCS, Lau PN, Au WL, Luo N (2008). Mapping the eight-item Parkinson’s disease questionnaire (PDQ-8) to the EQ-5D utility index. Qual Life Res.

[32] Brazier JE, Yang Y, Tsuchiya A, Rowen DL (2010). A review of studies mapping (or cross walking) non-preference based measures of health to generic preference-based measures. Eur J Health Econ.

[33] Pickard AS, Neary MP, Cella D (2007). Estimation of minimally important differences in EQ-5D utility and VAS scores in cancer. Health Qual Life Outcomes.

[34] Cheung YB, Thumboo J (2006). Developing health-related quality-of-life instruments for use in Asia: the issues. Pharmacoeconomics.

[35] Sakthong P, Charoenvisuthiwongs R, Shabunthom R (2008). A comparison of EQ-5D index scores using the UK, US, and Japan preference weights in a Thai sample with type 2 diabetes. Health Qual Life Outcomes.

